# Découverte fortuite d'une drépanocytose hétérozygote composite S/C

**DOI:** 10.11604/pamj.2017.27.93.12724

**Published:** 2017-06-07

**Authors:** Asmâa Biaz, Maroua Neji, Yousra Ajhoun, Samira EL Machtani Idrissi, Abdellah Dami, Karim Reda, Zohra Ouzzif, Sanae Bouhsain

**Affiliations:** 1 Service de Biochimie-Toxicologie Hôpital Militaire d’Instruction Mohammed V, Rabat, Maroc; 2 Service d’Ophtalmologie Hôpital Militaire d’Instruction Mohammed V, Rabat, Maroc; 3 Faculté de Médecine et de Pharmacie de Rabat, Université Mohamed V Souissi, Rabat, Maroc

**Keywords:** Rétinopathie, hémoglobinopathie, syndrome drépanocytaire composite SC, Retinopathy, hemoglobinopathy, composite S/C sickle cell disease

## Abstract

Le syndrome drépanocytaire composite SC représente 20% à 30% des syndromes drépanocytaires majeurs. Nous rapportons le cas d'une découverte fortuite d'une drépanocytose hétérozygote composite SC dans un contexte de décollement rétinien. Il s'agit d'une patiente hospitalisée au service d'ophtalmologie pour une baisse de l'acuité visuelle détectée depuis 06 mois et rebelle au traitement. Les antécédents cliniques sont dominés par la mise en place d'une prothèse totale de la hanche (PTH) douze ans auparavant. Notre observation rappelle la grande variabilité clinique de la maladie drépanocytaire imposant un dépistage précoce des patients à risque avec une surveillance clinique adaptée afin d'éviter l'évolution vers des séquelles organiques irréversibles telles que la rétinopathie drépanocytaire.

## Abstract (en)

Composite S/C sickle cell disease accounts for 20%-30% of major sickle cell syndromes. We report a case of fortuitous detection of composite heterozygous S/C sickle cell disease in the context of retinal detachment. The patient had been hospitalized in the Department of Ophthalmology for treatment-resistant decreased visual acuity detected 06 months before. The patient's clinical history was marked by total hip replacement (THR) twelve years before. Our study highlights the wide clinical variability of sickle cell disease underlying the importance of early screening and adapted clinical monitoring of patients at-risk, in order to avoid its evolution toward irreversible organic sequelae such as sickle cell retinopathy.

## Introduction

Le syndrome drépanocytaire composite SC représente 20% à 30% des syndromes drépanocytaires majeurs. Il est particulièrement fréquent dans certains pays de l'Afrique de l'Ouest tels que le Ghana, le Burkina Fasso, ou le Nigéria [1]. Il constitue une entité autonome très différente de la drépanocytose homozygote avec une atténuation des signes cliniques et une discrétion des anomalies hématologiques à l'origine d'un retard de diagnostic à l'âge adulte, au stade de séquelles irréversibles [2, 3]. Nous rapportons le cas d'une découverte fortuite d'une drépanocytose hétérozygote composite SC dans un contexte de décollement rétinien.

## Patient et observation

**Figure 1:**
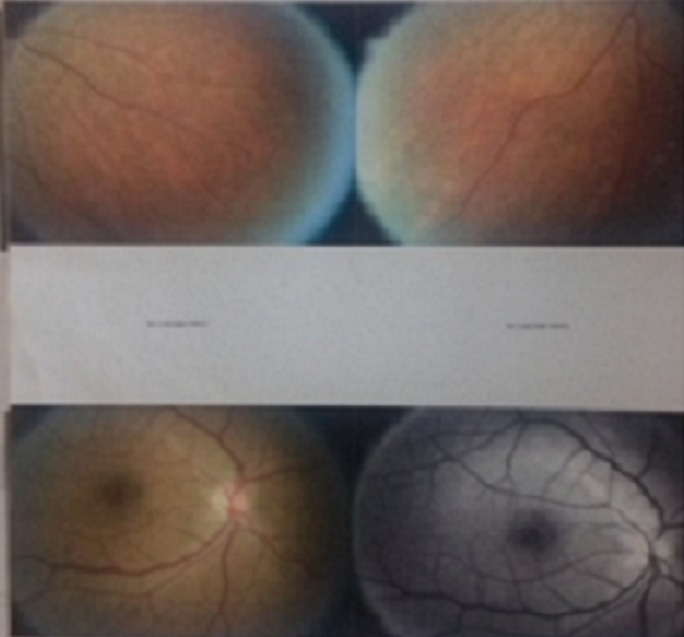
Fond d’œil

**Figure 2:**
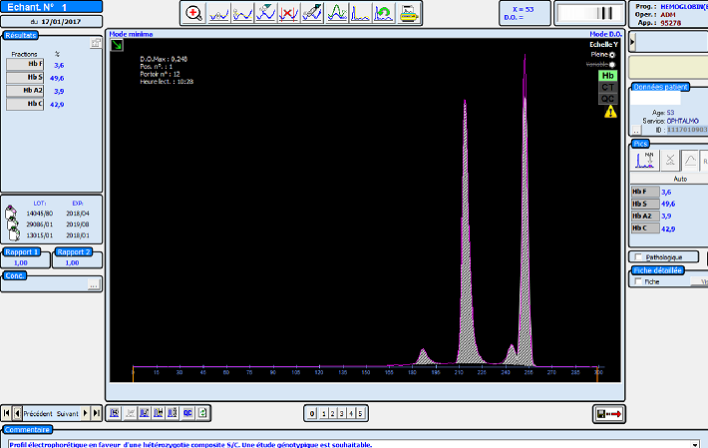
Profil électrophorétique de l’hémoglobine à pH alcalin

**Figure 3:**
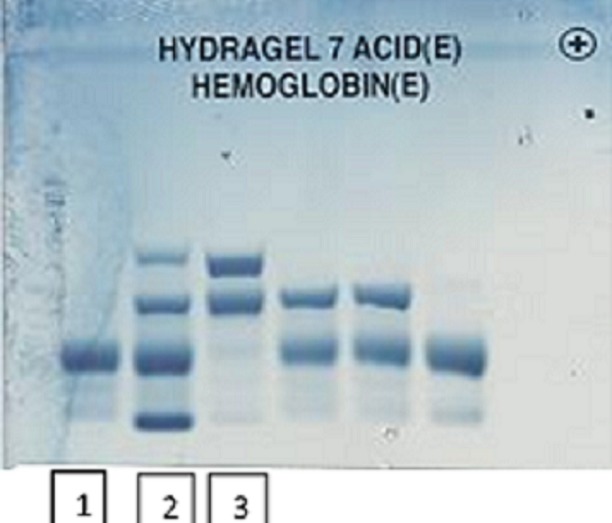
Electrophorèse de l’hémoglobine à pH acide: (1) contrôle normal; (2) contrôle pathologique AFSC; (3) patient

Il s'agit d'une patiente d'origine marocaine, âgée de 53 ans, mariée et mère de 03 enfants, hospitalisée au service d'ophtalmologie pour une baisse de l'acuité visuelle détectée depuis 06 mois et rebelle au traitement. Les antécédents cliniques sont dominés par la mise en place d'une prothèse totale de la hanche (PTH) douze ans auparavant. La patiente ne dispose cependant pas d'aucun compte rendu de cette hospitalisation. L'examen de fond dœil retrouve un décollement de la rétine de l'œil droit avec des signes de vascularite rétinienne bilatérale (rétinopathie stade IV) (Figure 1). L'hémogramme objective une discrète anémie avec un taux d'hémoglobine (Hb) à 11.8 g/dL normochrome normocytaire (volume globulaire moyen (VGM) à 89.2 fl et teneur corpusculaire moyenne en hémoglobine (TCMH) à 30.4 pg). L'examen du frottis sanguin suspecte la présence des hématies d'aspect en faucilles. Le test de falciformation est négatif. Le dosage de l'haptoglobine et des LDH est normal. Une électrophorèse capillaire de l'hémoglobine à pH alcalin est réalisée sur le système Capillarys 2 Flex piercing (Sebia^®^). Elle a révélé l'absence d'HbA, la présence d'Hb S à 49.6%, Hb C à 42.9%, HbA 2 à 3.9% et Hb F à 3.6% (Figure 2). L'électrophorèse de l'hémoglobine à pH acide sur gel d'agarose sur l'automate Hydrasys 2 Scan (Sebia^®^) confirme la double hétérozygotie SC (Figure 3). L'interrogatoire n'a pas retrouvé de notion de co-sanguinité des parents mais l'enquête familiale n'a pu être réalisée de même que l'étude génotypique. Le diagnostic retenu est un décollement rétinien compliquant une rétinopathie chez une patiente drépanocytaire hétérozygote composite SC. La prise en charge comprend un traitement par photocoagulation et une consultation spécialisée au service d'Hématologie Clinique en vue d'une prise en charge pluridisciplinaire.

## Discussion

Au Maroc, toute l'épidémiologie des hémoglobinopathies reste inconnue. L'OMS estime le taux des porteurs au Maroc à 6.5%; ce qui laisserait supposer l'existence de 30.000 cas de formes majeures de Thalassémie et Drépanocytose [4]. Au sein de notre institution, sur une période d'une année (résultats non publiés), sur les 246 études phénotypiques de l'hémoglobine réalisées, douze cas de drépanocytose ont été identifiés soit 4.87% dont deux soit 0.81% de drépanocytose hétérozygote composite SC. La drépanocytose hétérozygote composite SC est une entité autonome, très différente de la drépanocytose homozygote avec une maladie systémique moins sévère et moins invalidante. L'atténuation des symptômes cliniques et la discrétion des anomalies hématologiques font retarder le diagnostic à l'âge adulte. Chez notre patiente, seule une discrète anémie normochrome normocytaire a été retrouvée. L'évolution de la drépanocytose hétérozygote composite SC est cependant marquée par la survenue de séquelles irréversibles dont la physiopathologie fait intervenir essentiellement l'hyperviscosité. L'ostéo-nécrose aseptique de la hanche et la rétinopathie en sont les complications les plus fréquentes [5]. Une étude réalisée en Jamaïque portant sur 166 naissances dépistées drépanocytaires hétérozygotes SC et suivis à l'âge adulte a montré qu'une rétinopathie était diagnostiquée chez 43% d'entre eux [6]. Une autre étude portant sur le suivi de 106 patients SC, ayant un âge médian de 50 ans, a retrouvé comme principales complications les crises vaso-occlusives (65%), la rétinopathie (35%), l'ostéonécrose aseptique de hanche (23%) et les infarctus spléniques (19%) [7]. L'atteinte rétinienne serait en rapport avec les phénomènes vasoocclusifs entraînant l'apparition de zones non perfusées puis les stigmates de la rétinopathie proliférative apparaissent sous la forme de néovaisseaux fragiles qui se développent à la limite des zones normales et non perfusées. Les mécanismes exacts de cette vasculopathie sont mal connus mais certains facteurs de croissance vasculaires, comme le VEGF, ont été incriminés [8]. Chez notre patiente, ni la mise en place d'une PTH probablement secondaire à une ostéonécrose aseptique, ni les grossesses à risque n'ont permis de pousser les investigations pour poser le diagnostic du syndrome drépanocytaire composite, probablement en raison de la double discrétion des symptômes cliniques et des anomalies hématologiques. Une prise en charge précoce aurait cependant pu améliorer le pronostic fonctionnel et éviter la survenue du décollement rétinien.

## Conclusion

Notre observation rappelle la grande variabilité clinique de la maladie drépanocytaire imposant un dépistage précoce des patients à risque avec une surveillance clinique adaptée afin d'éviter l'évolution vers des séquelles organiques irréversibles telles que la rétinopathie drépanocytaire.

## Conflits d’intérêts

Les auteurs ne déclarent aucun conflit d'intérêts.
